# Nasopharyngeal tuberculosis: report of four cases and review of the literature

**DOI:** 10.11604/pamj.2019.33.150.15892

**Published:** 2019-06-27

**Authors:** Youssef Darouassi, Abdelfettah Aljalil, Amine Hanine, Amine Enneouali, Brahim Bouaity, Mohamed Mliha Touati, Haddou Ammar

**Affiliations:** 1 ENT Department, Military Hospital Avicenna, Marrakech, Morocco

**Keywords:** Tuberculosis, nasopharynx, treatment, nasopharyngeal neoplasm, nasopharyngeal disease, lymph node, headache, hearing loss, nasal obstruction

## Abstract

Even if tuberculosis is a major cause of morbidity and mortality, nasopharyngeal location is unusual and extremely rare. We report four new cases observed with short time interval suggesting a trend towards increased frequency. The diagnosis was confirmed by histological analysis after a biopsy. The evolution was favorable after anti tuberculosis chemotherapy. In the light of those observations and a review of the literature, we will discuss different characteristics of this disease and we will highlight the need of a systematic biopsy in order to confirm diagnosis and exclude undifferentiated carcinoma especially in endemic regions for both diseases.

## Introduction

Tuberculosis (TB) is a chronic bacterial infection due to *Mycobacterium tuberculosis*. It is one of the oldest human diseases, and still represents one of the major causes of morbidity and mortality worldwide. Approximately 9 million people contract TB and 2 million die of it yearly [1]. It can affect almost any organ, but nasopharyngeal location is unusual and extremely rare. In North Africa, the main diagnosis in case of nasopharyngeal swelling is undifferentiated carcinoma, hence the need of a systematic biopsy. We present four patients with nasopharyngeal TB and discuss different facets of this exceptional location.

## Patient and observation

### Case 1

**Figure 1:**
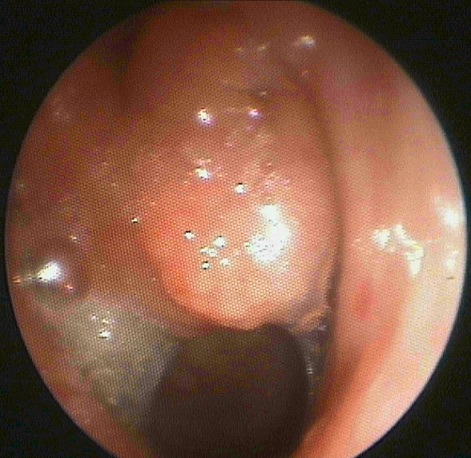
Endoscopique view showing a polypoid nasopharyngeal swelling in the first patient

**Figure 2:**
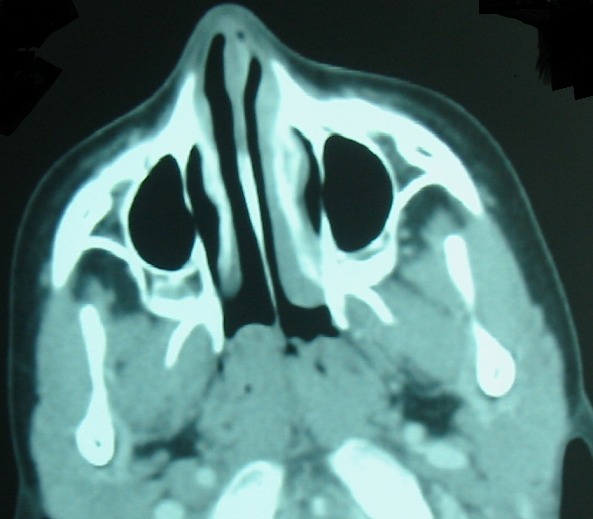
Computed tomography showing nasopharyngeal swelling in the first patient

The first patient was a 15-year-old boy with no family history of TB. He suffered chronic snoring since the age of six. He presented with headache, asthenia and weight loss. Examination on admission revealed multiple bilateral cervical lymphadenopathies in jugular areas. Nasal endoscopy (Figure 1) discovered a polypoid mass occupying the nasopharynx. A biopsy was performed. Computed Tomography (CT) showed a polypoid thickening of the posterior wall and the roof of the nasopharynx, measuring 2.5cm (Figure 2) with bilateral cervical lymphadenopathies in jugular areas.

### Case 2

**Figure 3:**
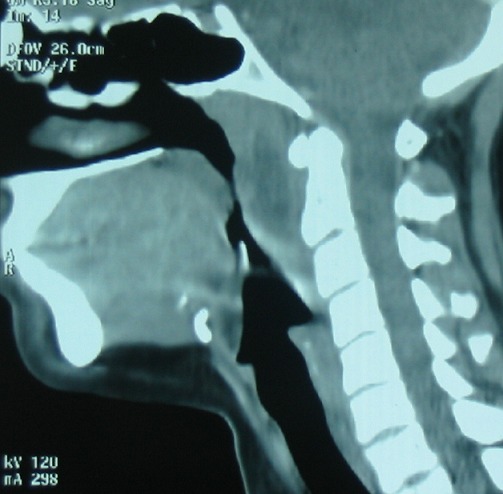
Computed tomography showing nasopharyngeal swelling with tissue necrosis in the second patient

The second patient was a 17-year-old girl without family history of TB. She presented with numerous bilateral cervical lymphadenopathies and nasal obstruction. Nasal endoscopy found a polypoid uprising of the superior and posterior nasopharyngeal walls. CT scan showed a nasopharyngeal mass with central hypodensity suggesting tissue necrosis (Figure 3), with multiple cervical lymphadenopathies. A biopsy was performed.

### Case 3

A 22-year-old man without past medical history was hospitalized for nasal obstruction with purulent rhinorrhea lasting for three months. Clinical examination found congestive nasal mucosa, purulent postnasal drip and a bilateral secretory otitis media with an average hearing loss of 25 dB, cervical palpation did not find any lymphadenopathy. Endoscopy objectified the presence of a tumor occupying substantially all of the nasopharynx, with irregular surface, covered with mucopurulent secretions and obstructing both choanae. CT scan showed a mass taking all the walls of the cavum without bone lysis. A biopsy was performed.

### Case 4

A 45 years old patient, chronic smoker, presented with unilateral lymphadenopathy gradually increasing in size with weight loss, associated with nasal obstruction and hearing loss of the right ear. Clinical examination found a painless firm lymphadenopathy of the right jugular era measuring 3cm and a secretory otitis media. The endoscopy found a tumor in the right posterolateral wall of the nasopharynx. CT scan showed a mass in the posterior wall of the nasopharynx with retropharyngeal lymphadenopathies. A biopsy was performed.

**Figure 4:**
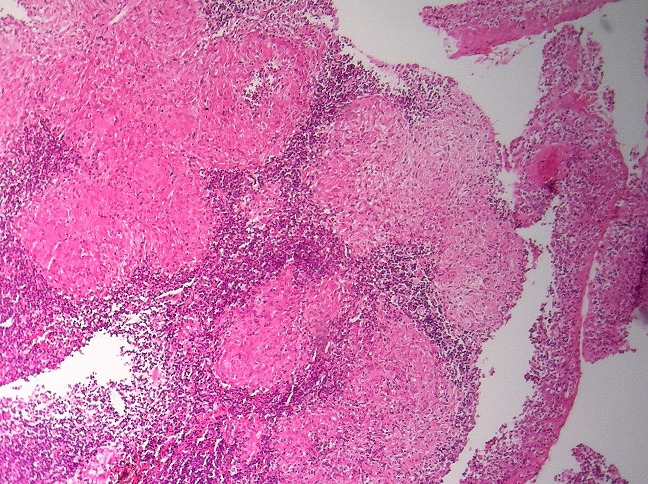
Histological section showing nasopharyngeal mucosa, epithelioid granulomas with giant cells and beginning of caseous necrosis

In four cases, histopathological study showed nasopharyngitis and granulomas with histiocytic, epithelioid, and giant multinucleated Langhans cells as well as caseous necrosis (Figure 4). Further examinations searching for other locations were negative confirming the diagnosis of primary nasopharyngeal tuberculosis. All patients received anti bacillary treatment based on rifampicin 10mg/kg/day, isoniazid 5mg/kg/day, pyrazinamide 20mg/kg/day during 2 months followed by rifampicin and isoniazid during 4 months. A clinical, endoscopic and histological surveillance was implemented and the evolution was favorable in all cases.

## Discussion

Up to 10% of TB cases have a presentation in head and neck region [2]. Cervical lymphadenitis is the most common followed by laryngeal TB [1]. Even if TB is more common in men, several recent studies have reported a higher incidence among women [1]. Advances in the areas of anti-bacillary chemotherapy, vaccination and pasteurization of milk reduced the incidence of pharyngeal TB until the mid-1980s, since then, other factors, such as human immunodeficiency virus (HIV) and the development of resistant strains seem to work in the opposite direction [3]. Although upper respiratory tract are the entry point of the *Mycobacterium tuberculosis*, their involvement is rare (less than 2%) [2]. Prevalence of TB lesions in the nose, mouth and pharynx, even at its peak in 1930 were reported in an average of 0.66% of cases [4]. Nasopharyngeal location is extremely rare (0.12% of all TB) and often primary; pulmonary and systemic involvement are unusual in those cases (25% to 30%) [2].

Lermoyes was the first to describe a nasopharyngeal TB in a 6-year-old child [3]. Since then, mostly isolated cases have been reported [2] most of them from endemic areas. Nasopharyngeal TB is often occult and found in children or young adults [3] as represented by our two first cases. Nasopharyngeal involvement is very unusual in pulmonary TB. In the other hand, throughout the rest of the upper aerodigestive tract (such as the larynx), pulmonary involvement is very frequent (95%), because of the spread by sputum from lung lesion [2].

TB infection can be contracted by inhalation, ingestion or inoculation. Inhalation is the major mode, ingestion has become rare due to the pasteurization of milk, as well as cutaneous inoculation [3,5]. Upper respiratory tract is resistant to TB, mainly because of the presence of saliva, which in addition to its cleansing action has an inhibitory effect on tubercle bacilli. Additional factors have contributed to this immunity against TB in this region including presence of saprophytes, the resistance of the striated musculature to bacterial invasion and the thickness of the mucosa [3-5]. However, even in the absence of mucosal breach, *Mycobacterium tuberculosis* can cross mucosal barriers by endocytosis in sites such as lymphoid pharyngeal tonsil, and phagocytes containing intracellular mycobacteria disseminate the infection [4].

Clinically, nasopharyngeal TB declares in 50% of cases by the presence of high jugular lymph nodes, followed by nasal symptoms (obstruction, rhinorrhea, snoring, postnasal drip) and otologic symptoms (hearing loss, tinnitus, otalgia); headache, sore throat and cough can also be observed [2,3,6-8]. Even diplopia was exceptionally reported [9,10]. Our first patient presented essentially with headache, the second presented with lymph nodes and nasal obstruction, the third suffered nasal obstruction with purulent rhinorrhea and hearing loss and the last one presented with lymph nodes, nasal obstruction and hearing loss. Endoscopy may show different aspects, ranging from apparently normal mucosa to an obvious mass, through aspects of a swollen, polyploid, ulcerated, leucoplasic mucosa and their various combinations [2]. But no endoscopic appearance is characteristic [3] and biopsy should be performed systematically. Neither CT scan nor magnetic resonance imaging shows typical appearance, but they may show signs of an inflammatory process [6]. Radiologically, there are two aspects: either polypoid mass or diffuse thickening of nasopharyngeal walls. Extension to prevertebral muscles and skull base is rare [2].

The diagnosis of pharyngeal TB is usually confirmed by histological examination of biopsy and culture on specific environment [3]. PCR (polymerase chain reaction) of bacterial Deoxyribonucleic acid (DNA) and microscopic examination of cultures facilitate the diagnosis [1,11]. Histologically, the presence of epithelioid granulomas with giant cells and caseous necrosis is pathognomonic [12]. Weller classified nasopharyngeal TB granulomas into 3 types: submucosal, in lymphoid follicles or mixed [3]. Differential diagnosis includes sarcoidosis, Wagener disease, fungal infections, reactions to carcinoma, Hodgkin's lymphoma (in particular), radiotherapy, syphilis, leprosy, periartiritis nodosa and nasopharyngeal carcinoma [2,3,6]. Association of nasopharyngeal carcinoma with TB is possible but exceptional [13,14].

Once diagnosis of pharyngeal TB is confirmed, search for other locations is essential to adjust the treatment. Seeking HIV should be systematic [12]. The treatment of nasopharyngeal TB is based on antituberculous chemotherapy (rifampicin, isoniazid, ethambutol, pyrazinamide and streptomycin) during 6 months, up to 24 months in some resistant forms. The evolution is usually favorable. However, sequels are possible in case of velar or tonsillar involvement such as perforations, adhesions and retractile scars with more or less functional troubles which may require surgical treatment [12].

## Conclusion

We tend to believe that the frequency of rhinopharyngeal TB is increasing but it is difficult to prove because of the lack of data in the literature. This diagnosis should be considered in endemic areas and in immunocompromised patients. Clinical symptoms and radiological findings are not specific, and it is difficult to distinguish from undifferentiated carcinoma, particularly in areas where both are endemic, such as in North Africa, which makes necessary to perform a biopsy of every unusual nasopharyngeal aspect for histological but also bacteriological analysis. Association of nasopharyngeal carcinoma and TB is possible but rare.

## Competing interests

The authors declare no competing interests.
